# Hydrogen Sulfide Increases the Analgesic Effects of µ- and δ-Opioid Receptors during Neuropathic Pain: Pathways Implicated

**DOI:** 10.3390/antiox11071321

**Published:** 2022-07-04

**Authors:** Xue Bai, Gerard Batallé, Gianfranco Balboni, Olga Pol

**Affiliations:** 1 Grup de Neurofarmacologia Molecular, Institut d’Investigació Biomèdica Sant Pau (IIB Sant Pau), 08041 Barcelona, Spain; xue.bai@e-campus.uab.cat (X.B.); gerard.batalle@e-campus.uab.cat (G.B.); 2 Grup de Neurofarmacologia Molecular, Institut de Neurociències, Universitat Autònoma de Barcelona, 08193 Barcelona, Spain; 3 Unit of Pharmaceutical, Pharmacological and Nutraceutical Sciences, Department of Life and Environmental Sciences, University of Cagliari, 09042 Cagliari, Italy; gbalboni@unica.it

**Keywords:** analgesia, apoptosis, hydrogen sulfide, neuropathic pain, opioids, oxidative stress

## Abstract

Recent studies have revealed that hydrogen sulfide (H_2_S) increases the analgesic actions of the δ-opioid receptor (DOR) in inflammatory pain. However, the possible improvement of the analgesia of μ-opioid receptor (MOR) and DOR agonists during neuropathic pain, through pretreatment with two slow-releasing H_2_S donors—DADS (diallyl disulfide) and GYY4137 (morpholin-4-ium 4-methoxyphenyl(morpholino) phosphinodithioate dichloromethane complex)—is still unknown. In male C57BL/6J mice with neuropathic pain incited by chronic constriction of the sciatic nerve (CCI), we evaluated: (1) the influence of DADS (3.5 mg/kg) and GYY4137 (0.7 mg/kg) on the inhibition of the allodynia and hyperalgesia produced by the systemic or local administration of morphine (3 mg/kg or 65 µg) and UFP-512 (1 mg/kg or 12.5 µg); (2) the reversion of the antinociceptive actions of high doses of DADS (30 mg/kg) and GYY4137 (24 mg/kg) with MOR and DOR antagonists; and (3) the effects of H_2_S donors on oxidative stress, apoptotic responses, and MOR and DOR expression in the medial septum (MS) and dorsal root ganglia (DRG). The results revealed that both DADS and GYY4137 improved the antiallodynic effects of morphine and UFP-512, possibly by up-regulating MOR and DOR expression in DRG. The administration of MOR and DOR antagonists blocked the analgesic properties of DADS and GYY4137, revealing the feasible participation of the endogenous opioid system in H_2_S analgesic effects. Moreover, both H_2_S donors inhibited oxidative stress and apoptosis generated by CCI in the MS and/or DRG. This study suggests the co-treatment of H_2_S donors with MOR or DOR agonists as a potential therapy for neuropathic pain.

## 1. Introduction

Neuropathic pain has a high prevalence (6.9–10%), and is one of the most common clinical symptoms [1]. Neuropathic pain can be caused by a variety of etiologies, some of which are common, such as nerve injury, trauma, drugs, infections. Neuropathic pain can be also linked with several metabolic disorders—for example, diabetes and neurodegenerative diseases such as Parkinson and Alzheimer [2,3]. Neuropathic pain is characterized by different degrees of allodynia and hyperalgesia that severely affect the patient’s quality of life. This type of pain is difficult to treat, given the variety and complexity of its manifestations, and the multiple adverse effects accompanying the current pharmacological treatments [4,5].

Oxidative stress, resulting from the increased production of reactive oxygen species (ROS) and the disruption of redox balance caused by damage [6], is an important pathophysiological process following nerve injury, and is deeply involved in the development of neuropathic pain. Moreover, excessive production of ROS and oxidative stress causes abnormal mitochondrial structure, leading to mitochondrial dysfunction and apoptosis [7]. Several investigations have confirmed that mitochondrial dysfunction is an important cause of neurodegenerative diseases such as Alzheimer, Parkinson, and Huntington [8,9], and most of these diseases lead to motor neuron dysfunctions that in turn induce chronic pain [10,11,12].

Opioids are among the most-used analgesics in clinical practice, although µ-opioid receptor (MOR) agonists are relegated to third line clinically recommended treatments for neuropathic pain, due to their low efficacy and common undesirable side-effects, such as respiratory depression, constipation, addiction, and tolerance [13,14]. Consequently, several studies have demonstrated the low palliative efficacy of morphine in different preclinical models of neuropathic pain [15,16], and have revealed that the effectiveness of morphine in inhibiting neuropathy was much lower than it was for inhibiting inflammatory pain [16]. Interestingly, treatment with DOR agonists reduced chronic neuropathic [17,18] and inflammatory pain in rodents [19,20] with similar effectiveness, although its efficacy was moderate [16,18]. Thus, additional therapeutic strategies are needed to potentiate the efficacy of opioids for neuropathic pain.

Hydrogen sulfide (H_2_S) is a gaseous neurotransmitter, widely distributed in the central (CNS) and peripheral (PNS) nervous systems, which modulates several physiological and pathological processes [21,22]. Recent preclinical studies have shown that treatment with two slow releasers of H_2_S, a component of garlic, diallyl disulfide (DADS), and a synthetic H_2_S donor, GYY4137 (morpholin-4-ium 4-methoxyphenyl(morpholino) phosphinodithioate dichloromethane complex), relieved neuropathic [23] and osteoarthritic pain [24]. The analgesic actions of both compounds were mainly produced by inhibiting apoptotic responses, and activating the endogenous antioxidant system by triggering the synthesis of antioxidant enzymes—such as superoxide dismutase 1 (SOD-1), glutathione S-transferase Mu 1 (GSTM1), heme oxygenase 1 (HO-1), and quinone oxidoreductase 1 (NQO1)—in the amygdala (AMG) and periaqueductal gray matter (PAG) of mice with nerve-injury-induced neuropathy [23]. Nonetheless, DADS and GYY4137 actions in DRG—one of the initial parts of the ascending transmission pathways involved in the development and maintenance of neuropathic pain [25,26]—and in other brain areas involved in pain modulation, such as the medial septum (MS) [27,28], have not been previously evaluated.

In addition, a recent study proved that the administration of slow-releasing H_2_S donors improved the antinociceptive effects of DOR agonists [D-Pen2, D-Pen5]-enkephalin, and H-Dmt-Tic-NH-CH(CH2-COOH)-Bid (UFP-512) during inflammatory pain, by enhancing the peripheral expression of the DOR [20]. Thus, the co-administration of H_2_S donors with DOR agonists is a potential therapy for inflammatory pain. However, the possible potentiation of the analgesic properties of MOR and DOR agonists with their co-treatment with DADS and GYY4137 during neuropathic pain, and the possible mechanisms involved, are still unknown.

Therefore, in animals with neuropathic pain produced by the chronic constriction of the sciatic nerve (CCI), we evaluated: (1) the impact of the co-administration of DADS and GYY4137 with MOR (morphine) and DOR (UFP-512) agonists in the allodynic and hyperalgesic responses provoked by nerve injury; (2) the reversion of H_2_S donor effects by MOR (naloxone) and DOR (naltrindole) antagonists; and (3) the influence of DADS and GYY4137 treatments on the oxidative stress, apoptosis, and protein levels of MOR and DOR in the MS and DRG.

## 2. Materials and Method

### 2.1. Animals

These studies were conducted with male C57BL/6 mice (21–26 g; 5–6 weeks old), purchased from Envigo Laboratories (Barcelona, Spain), which were accommodated under normal light/dark (12/12-h), temperature (22 °C), and humidity (66%) conditions, with free access to food and water. The experiments were conducted after 7 days of acclimatization to the environment. The experiments were performed between 9:00 a.m. and 5:00 p.m. in compliance with the guidelines of European Commission Directive (2010/63/EC) and the Spanish Law (RD 53/2013) regulating animal research, and were approved by the local Committee of Animal Use and Care of the Autonomous University of Barcelona, Barcelona, Spain (ethical code 1319). The greatest efforts were made to minimize the number of animals employed, and their suffering.

### 2.2. Induction of Neuropathic Pain

The CCI performed under isoflurane anesthesia conditions (3% induction, 2.5% maintenance) was used as a model of neuropathic pain. After separation of the biceps femoris and the gluteus superficialis by blunt dissection, three ligatures right (4/0 silk) around the sciatic nerve were performed, taking care to maintain epineural circulation [29]; identical conditions were applied to control animals without nerve ligation (SHAM).

### 2.3. Mechanical Allodynia

Mechanical allodynia was evaluated by measuring hind paw withdrawal response after stimulation by von Frey filaments of different bending forces (0.4–3.5 g). Mice were put in Plexiglas tubes (20 cm high × 9 cm diameter) with a wire grid bottom, through which the filaments (North Coast Medical, Inc., San Jose, CA, USA) were applied by using the up–down paradigm [30]. Filaments of 0.4 and 3.5 g were used first and last, respectively. The strength of the following filament was increased or decreased depending on the animal’s response. The threshold of the response was calculated using an Excel program (Microsoft Iberia SRL, Barcelona, Spain) that included curve fitting of the data.

### 2.4. Thermal Hyperalgesia

Thermal hyperalgesia was evaluated by measuring paw withdrawal latency in response to radiant heat in the plantar test (Ugo Basile, Varese, Italy) [31]. Mice were placed in Plexiglas tubes (20 cm high × 9 cm diameter) placed on a glass surface. The heat source positioned under the plantar surface of the hind paws was activated with a light beam intensity until the paw withdrawal. A cut-off time of 12 s was used to prevent paw damage. Mean paw-withdrawal latencies were determined from the average of three separate trials.

### 2.5. Cold Allodynia

A cold plate apparatus (Ugo Basile, Varese, Italy) was used for evaluating cold allodynia. The number of elevations of each hind paw of animals exposed to the cold plate (4 ± 0.5 °C) for 5 min, was recorded.

In all tests, the animals were habituated to the environment for 1 h before the experiment. Both the ipsilateral and the contralateral hind paws were tested, and the experiments were performed by experimenters blinded to the experimental conditions.

### 2.6. Western Blot Analysis

The animals were euthanized by cervical dislocation at 30 days after surgery (CCI or SHAM). The MS and ipsilateral DRG were extracted immediately, rapidly frozen in liquid nitrogen, and preserved at −80 °C until assay. The sonication of tissues was made in cold lysis RIPA Buffer (Sigma–Aldrich, St Louis, MO, USA), and after their solubilization for 1 h at 4 °C, the crude homogenates were sonicated for 10 s and centrifuged (700 g) for 20 min at 4 °C. The supernatant (60 μg of total protein) was mixed with 4X Laemmli loading buffer and loaded onto 4% stacking/12% separating sodium dodecyl sulfate polyacrylamide gels. Proteins were electrophoretically transferred onto a polyvinylidene fluoride membrane for 120 min, and blocked with phosphate-buffered saline (PBS; P-5493; Sigma–Aldrich, St. Louis, MO, USA) containing non-fat dry milk (5%), Tris-buffered saline with Tween 20 containing bovine serum albumin (5%) (BSA; Sigma–Aldrich, St. Louis, MO, USA), or non-fat dry milk (5%) and PBS with Tween 20 containing BSA (5%), for 75 min. Then, they were incubated overnight at 4 °C with specific rabbit primary antibodies: anti HO-1 (1:150; Enzo Life Sciences, New York, NY, USA); NQO1 (1:200; Sigma–Aldrich, St. Louis, MO, USA); SOD-1 (1:150; Novus Biologic, Littleton, CO, USA); GSTM1 (1:150; Novus Biologic, Littleton, CO, USA); 4-HNE (1:200; Abcam, Cambridge, UK); BAX (1:250; Cell Signaling Technology, Danvers, MA, USA); MOR (1: 300; Abcam, Cambridge, UK); DOR (1:300; Abcam, Cambridge, UK); or glyceraldehyde-3-phosphate dehydrogenase (GAPDH; 1:5000, Merck, Billerica, MA, USA). The blots were then incubated with anti-rabbit secondary polyclonal antibodies conjugated to horseradish peroxidase (GE Healthcare, Little Chalfont, Buckinghamshire, UK) for 1 h at room temperature. The proteins were detected by utilizing chemiluminescence reagents provided in an ECL kit (GE, Healthcare, Little Chalfont, Buckinghamshire, UK). Densitometric analysis was carried out using the Image-J program (National Institutes of Health, Bethesda, MD, USA).

### 2.7. Experimental Procedures

To investigate the effects of H_2_S in the analgesic actions of MOR and DOR agonists, the inhibition of mechanical and cold allodynia, and thermal hyperalgesia produced by low doses of DADS (3.5 mg/kg) or GYY4137 (0.7 mg/kg) intraperitoneally injected in combination with the i.p. or subplantar (s.p.) injection of low doses of morphine (3 mg/kg, i.p.; 65 µg, s.p.) or UFP-512 (1 mg/kg, i.p.; 12.5 µg, s.p.), were evaluated. The local UFP-512 dose was extracted from the response curve executed in this study (Figure S1), and the intraperitoneally and/or subplantarly injected doses of UFP-512, morphine, DADS, and GYY4137 were selected in accordance with previous studies [18,23,29,32]. DADS and GYY4137 were injected 30 min before morphine or UFP-512 injections, and tests were conducted 30 min later (*n* = 6 animals for group).

In other experiments, we assessed the reversion, by the i.p. or s.p. administration of the MOR antagonist (naloxone, 3 mg/kg; 20 µg) or the DOR antagonist (naltrindole, 3 mg/kg; 50 µg), of the effects produced by high doses of DADS (30 mg/kg) or GYY4137 (24 mg/kg). DADS and GYY4137 were injected 15 min before naloxone or naltrindole administration, and tests were conducted 60 min later (*n* = 6 animals per group). The doses of DADS, GYY4137, naloxone, and naltrindole were selected in accordance with previous studies [18,23,29,32]. In all experiments, saline (SS, 0.4% NaCl) plus SS-treated animals were used as controls.

Finally, nerve-injured mice injected with DADS or GYY4137 were euthanized by cervical dislocation, and the HO-1, NQO1, SOD-1, GSTM1, 4-HNE, BAX, MOR, and DOR protein levels in the MS and DRG were assessed by Western blot. Sham-operated animals injected with SS were utilized as controls (*n* = 3 samples).

### 2.8. Drugs

DADS and GYY4137, purchased from Sigma–Aldrich (St. Louis, MO, USA), were dissolved in SS, and intraperitoneally injected in a final volume of 10 mL/kg at 1 h, before testing in compliance with other works [23,33].

Morphine hydrochloride purchased from Alcaiber S.A. (Madrid, Spain), UFP-512 synthesized by [34], and naloxone and naltrindole obtained from Sigma–Aldrich (St. Louis, MO, USA), were dissolved in SS and intraperitoneally or subplantarly injected in a final volume of 10 mL/kg or 30 µL, in agreement with previous studies [18,23,29,32].

All drugs were prepared just before use. For each group injected with a drug, the corresponding control group received the identical volume of SS.

### 2.9. Statistical Analyses

Data are expressed as the mean values ± standard error of the mean (SEM). GraphPad software (version 8.0) was used to perform the statistical analysis. In each behavior test, the evaluation of the effects of different doses of UFP-512 or SS was performed by using a one-way analysis of variance (ANOVA) followed by the Tukey test. A one-way ANOVA followed by the Tukey test was also applied to evaluate the effects of DADS and GYY4137 injected alone or mixed with morphine, UFP-512, naloxone or naltrindole.

The effects of the H_2_S donors on the expression of several proteins were also analyzed by using a one-way ANOVA and the post hoc Tukey test.

A value of *p* < 0.05 was considered significant.

## 3. Results

### 3.1. The Effects of the Co-Administration of DADS or GYY4137 with Morphine or UFP-512 on the Allodynia and Hyperalgesia Caused by Nerve Injury

In mice with neuropathic pain induced by CCI, we assessed the mechanical and cold antiallodynic effects, as well as the antihyperalgesic effects induced by the i.p. administration of a low dose of DADS (3.5 mg/kg) or GYY4137 (0.7 mg/kg) combined with low doses of morphine (3 mg/kg or 65 µg) or UFP-512 (1 mg/kg or 12.5 µg), intraperitoneally or subplantarly injected. The effects of each of these treatments administered alone were also assessed.

**Figure 1:**
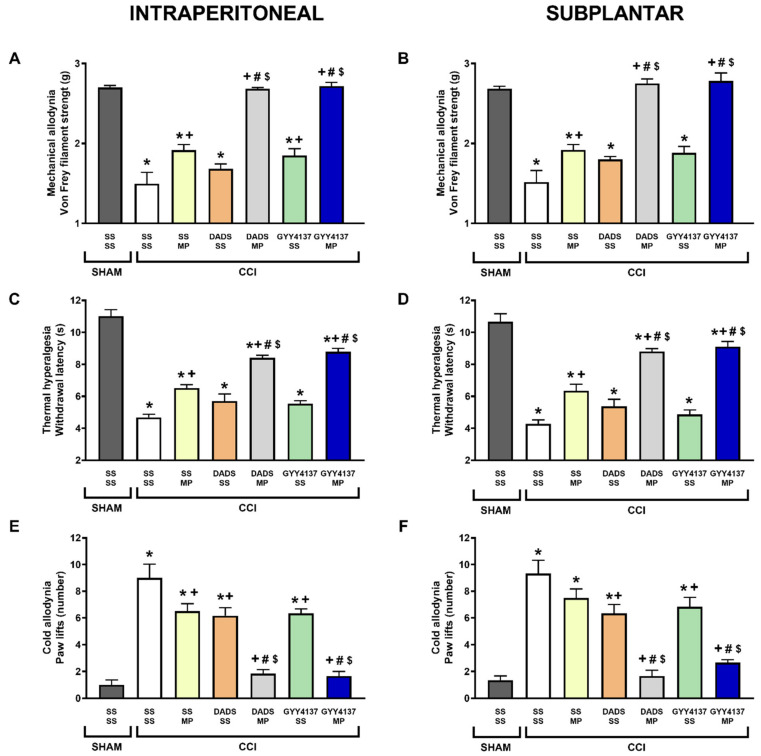
Effects of the co-administration of DADS or GYY4137 with morphine on the mechanical allodynia, thermal hyperalgesia, and cold allodynia induced by nerve injury in mice. Effects are represented of the i.p. administration of 3.5 mg/kg of DADS or 0.7 mg/kg of GYY4137 combined with 3 mg/kg or 65 µg of morphine (MP), intraperitoneally or subplantarly injected, on the mechanical allodynia (**A**,**B**), thermal hyperalgesia (**C**,**D**), and cold allodynia (**E**,**F**) provoked by sciatic nerve injury (CCI) in the ipsilateral paws. The effects of these treatments administered alone are also represented. Sham-operated (SHAM) animals treated with SS plus SS are also represented. For each test, * represents significant differences vs. SHAM-animals treated with SS plus SS, + vs. CCI-mice treated with SS plus SS, # vs. CCI-mice treated with SS plus MP and $ vs. CCI-mice treated with DADS or GYY4137 plus SS (*p* < 0.05; one-way ANOVA, followed by Tukey test). Each column represents the mean, and vertical bars indicate SEM (*n* = 6 animals for treatment).

Our results confirmed that CCI reduced the threshold of ipsilateral hind paw withdrawal from von Frey filaments stimulation (*p* < 0.001, one-way ANOVA followed by the Tukey test vs. sham-operated mice treated with SS plus SS; Figure 1A,B), as well as the withdrawal threshold of the ipsilateral hind paw in response to a thermal stimulus (*p* < 0.001, one-way ANOVA followed by the Tukey test vs. sham-operated mice treated with SS plus SS; Figure 1C,D), and increased the number of ipsilateral hind paw lifts caused by cold stimulus at day 30 after surgery (*p* < 0.001, one-way ANOVA followed by Tukey test; vs. sham-operated mice treated with SS plus SS; Figure 1E,F).

Moreover, treatment with DADS or GYY4137 did not alter but slightly inhibited the mechanical allodynia (Figure 1A,B), thermal hyperalgesia (Figure 1C,D), and cold allodynia (Figure 1E,F) provoked by nerve injury (*p* < 0.001; one-way ANOVA vs. CCI- SS plus SS treated mice). Co-administration of both H_2_S donors with morphine increased the mechanical antiallodynic (Figure 1A,B), thermal antihyperalgesic (Figure 1 C,D), and cold antiallodynic (Figure 1E,F) effects produced by the i.p. and s.p. injection of morphine in the ipsilateral paw of nerve-injured mice (*p* < 0.001, one-way ANOVA followed by Tukey test; vs. their respective control group treated with SS plus SS or morphine, and with mice treated with DADS or GYY4137 plus SS). Nevertheless, while the co-administration of both H_2_S donors with morphine completely blocked the mechanical and cold allodynia, thermal hyperalgesia was only significantly reduced (*p* < 0.001; one-way ANOVA vs. sham-operated mice treated with SS plus SS), thus revealing that the co-administration of H_2_S plus morphine, systemically or locally administered, was more effective in inhibiting allodynia than hyperalgesia.

**Figure 2:**
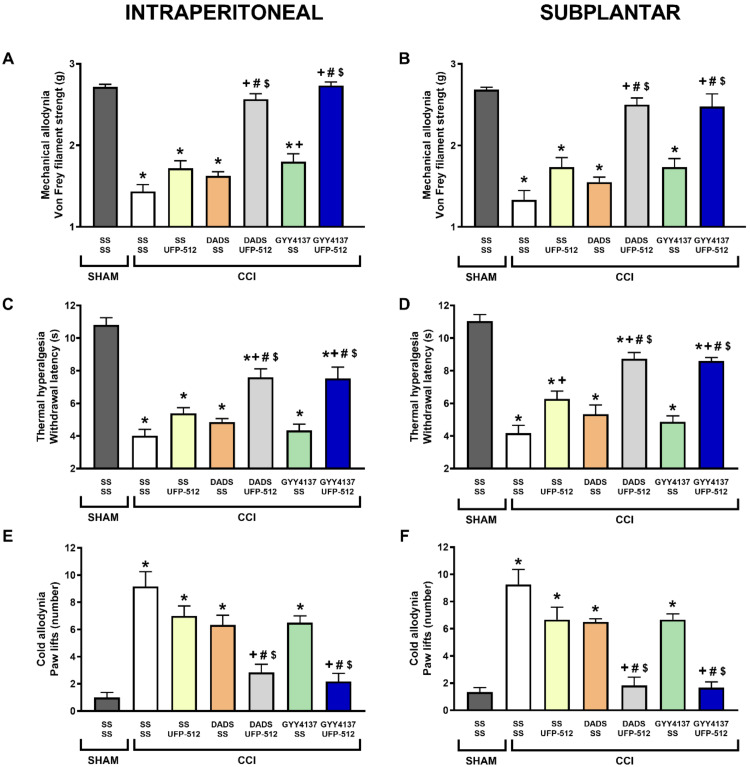
Effects of the co-administration of DADS or GYY4137 with UFP-512 on the mechanical allodynia, thermal hyperalgesia, and cold allodynia induced by nerve injury in mice. Effects are represented of the i.p. injection of DADS (3.5 mg/kg) or GYY4137 (0.7 mg/kg) co-administered with 1 mg/kg or 12.5 µg of UFP-512, intraperitoneally or subplantarly injected, on the mechanical allodynia (**A**,**B**), thermal hyperalgesia (**C**,**D**), and cold allodynia (**E**,**F**) provoked by sciatic nerve injury (CCI) in the ipsilateral paws. The effects of these treatments administered alone are also represented. Sham-operated (SHAM) animals treated with SS plus SS are also represented. For each test, * represents significant differences vs. SHAM-animals treated with SS plus SS, + vs. CCI-mice treated with SS plus SS, # vs. CCI-mice treated with SS plus UFP-512 and $ vs. CCI-mice treated with DADS or GYY4137 plus SS (*p* < 0.05; one-way ANOVA, followed by Tukey test). Each column represents the mean, and vertical bars indicate SEM (*n* = 6 animals for treatment).

Our results also demonstrated that treatment with 3.5 mg/kg of DADS or 0.7 mg/kg of GYY4137 significantly enhanced the mechanical antiallodynic (Figure 2A,B), thermal antihyperalgesic (*p* < 0.001, one-way ANOVA; Figure 2C,D), and cold antiallodynic effects (*p* < 0.001, one-way ANOVA; Figure 2E,F) produced by the i.p. (1 mg/kg) or s.p. (12.5 µg) administration of UFP-512 (*p* < 0.001, one-way ANOVA, followed by Tukey test; vs. their respective control group treated with SS plus SS or UFP-512, and with mice treated with DADS or GYY4137 plus SS).

As with the morphine, whereas mechanical and cold allodynia were completely blocked with the co-administration of both H_2_S donors with UFP-512, thermal hyperalgesia was not totally reduced (*p* < 0.001; one-way ANOVA vs. sham-operated mice treated with SS plus SS), thus revealing that the co-administration of H_2_S donors plus UFP-512, systemically or locally administered, was more effective in inhibiting mechanical and cold allodynia than thermal hyperalgesia provoked by nerve injury.

Lastly, DADS and GYY4137 administered alone, or combined with the i.p. and s.p. injection of morphine or UFP-512, did not have any effect in the contralateral paws of sciatic nerve-injured animals, nor in the contralateral and ipsilateral paws of sham-operated treated animals (data not displayed).

### 3.2. Reversal of the Antinociceptive Effects of DADS and GYY4137 with MOR and DOR Antagonists

To investigate the possible involvement of the endogenous opioid system in the analgesic actions of DADS and GYY4137 during neuropathic pain, we assessed the reversion of the antinociceptive actions produced by a high dose of DADS (30 mg/kg) or GYY4137 (24 mg/kg) with the administration of the MOR antagonist, naloxone, injected at 3 mg/kg, i.p. or 20 µg, s.p. The effects induced by each of these treatments administered individually were also evaluated.

**Figure 3:**
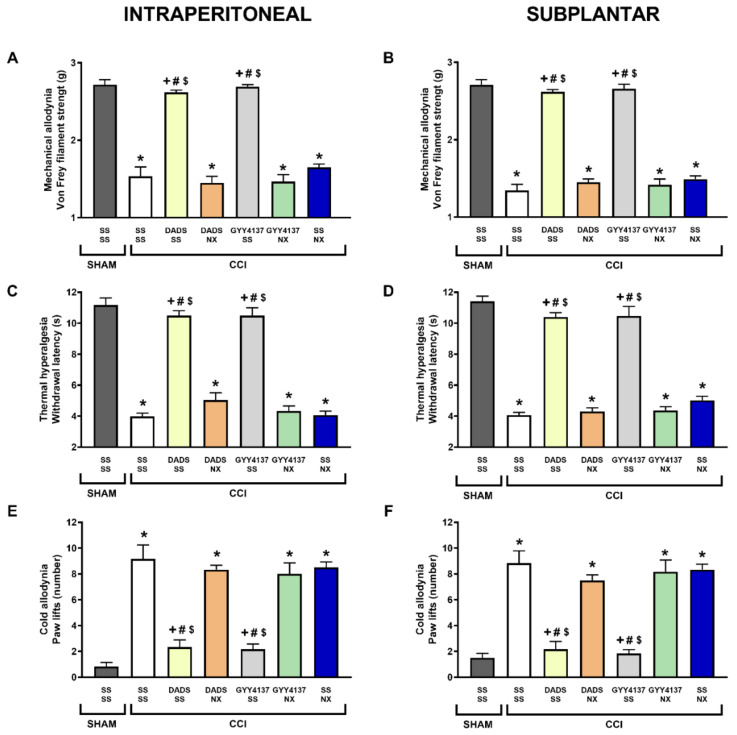
Reversal of the antiallodynic and antihyperalgesic effects of DADS and GYY4137 by naloxone during neuropathic pain. The inhibitory actions produced by i.p. injection of DADS (30 mg/kg) or GYY4137 (24 mg/kg) alone, and combined with naloxone (NX) intraperitoneally (3 mg/kg) (**A**,**C**,**E**) or subplantarly injected (20 µg) (**B**,**D**,**F**) in mice with CCI-provoked neuropathic pain, are represented. Sham-operated (SHAM) animals treated with SS plus SS are also represented. For each test, * represents significant differences vs. SHAM-animals treated with SS plus SS, + vs. CCI-mice treated with SS plus SS, # vs. CCI-mice treated with DADS or GYY4137 plus NX, and $ vs. CCI-mice treated with SS plus NX (*p* < 0.05; one-way ANOVA, followed by Tukey test). Each column represents the mean, and vertical bars indicate SEM (*n* = 6 animals for treatment).

Our results demonstrated that systemic and local treatment with naloxone reversed the inhibition of the mechanical allodynia (*p* < 0.001, one-way ANOVA; Figure 3A,B), thermal hyperalgesia (*p* < 0.001, one-way ANOVA; Figure 3C,D) and cold allodynia (*p* < 0.001, one-way ANOVA; Figure 3E,F) induced by both H_2_S donors. In all tests, i.p. or s.p. treatment with naloxone did not alter the nociceptive responses induced by CCI.

**Figure 4:**
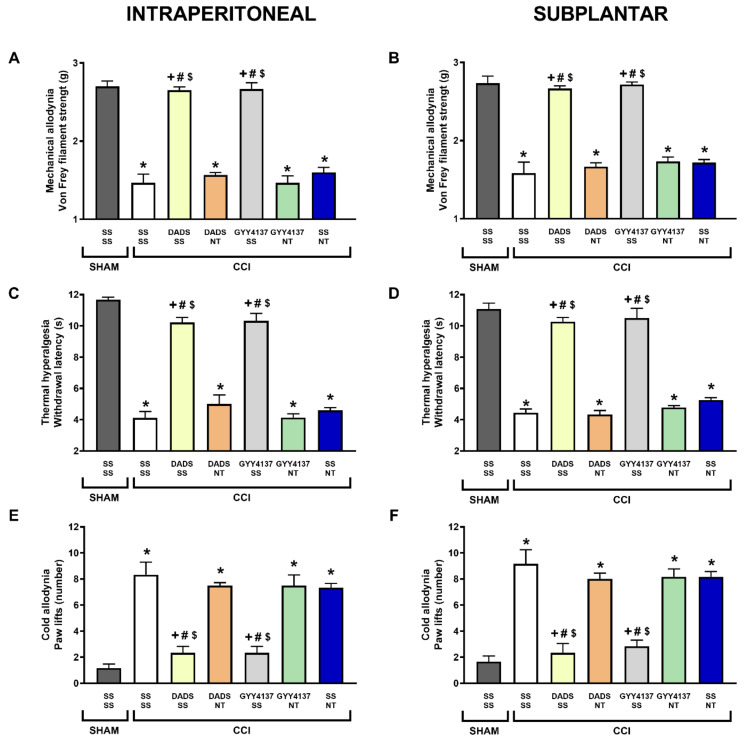
Reversal of the antiallodynic and antihyperalgesic effects of DADS and GYY4137 by naltrindole during neuropathic pain. The inhibitory actions induced by the i.p. injection of DADS (30 mg/kg) or GYY4137 (24 mg/kg) alone, and combined with naltrindole (NT) intraperitoneally (3 mg/kg) (**A**,**C**,**E**) or subplantarly injected (50 µg) (**B**,**D**,**F**) in mice with CCI-provoked neuropathic pain, are represented. Sham-operated (SHAM) animals treated with SS plus SS are also represented. For each test, * represents significant differences vs. SHAM-animals treated with SS plus SS, + vs. CCI-mice treated with SS plus SS, # vs. CCI-mice treated with DADS or GYY4137 plus NT, and $ vs. CCI-mice treated with SS plus NT (*p* < 0.05; one-way ANOVA, followed by Tukey test). Each column represents the mean, and vertical bars indicate SEM (*n* = 6 animals for treatment).

Similar results were obtained with the co-administration of DADS (30 mg/kg) or GYY4137 (24 mg/kg) with the DOR antagonist, naltrindole. Indeed, the i.p. (3 mg/kg) or s.p. (50 µg) injection of naltrindole reversed the inhibition of the mechanical allodynia (*p* < 0.001, one-way ANOVA; Figure 4A,B), thermal hyperalgesia (*p* < 0.001, one-way ANOVA; Figure 4C,D), and cold allodynia (*p* < 0.001, one-way ANOVA; Figure 4E,F) produced by high doses of DADS and GYY4137. In all tests, i.p. or s.p. treatment with naltrindole did not alter the nociceptive responses induced by CCI.

Finally, the administration of SS, DADS, or GYY4137 alone, and combined with naloxone or naltrindole, did not alter the responses obtained in the contralateral paws of nerve-injured animals, nor in the contralateral and ipsilateral paws of sham-operated mice (data not displayed).

### 3.3. Effects of DADS and GYY4137 on the Expression of Antioxidant Enzymes in the MS and DRG of Nerve-Injured Mice

**Figure 5:**
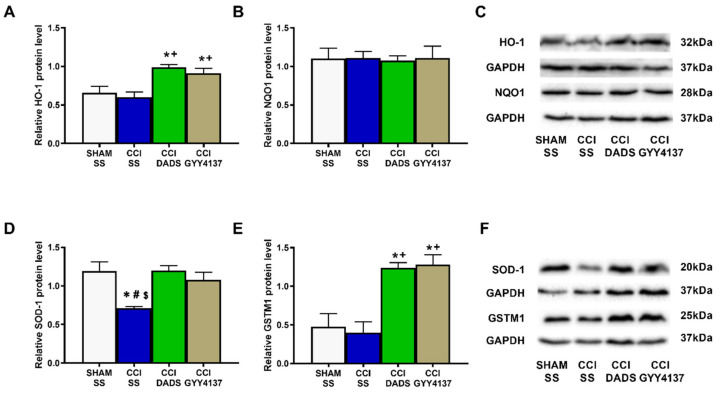
The influence of DADS and GYY4137 treatments on the expression of HO-1, NQO1, SOD-1, and GSTM1 in the MS of nerve-injured mice. Both treatments improved the HO-1 (**A**) and GSTM1 (**E**) levels, and normalized the downregulation of SOD-1 (**D**) induced by CCI. No alterations in the expression of NQO1 (**B**) were identified. Sham-operated (SHAM) mice treated with SS were used as control groups. All proteins are expressed relative to GAPDH levels. Blots for HO-1 and NQO1 (**C**), as well as for SOD-1 and GSTM1 (**F**), are displayed. In all pictures, * denotes significant differences vs. SHAM-animals treated with SS; + vs. CCI-mice treated with SS; # vs. CCI-mice treated with DADS; and $ vs. CCI-mice treated with GYY4137 (*p* < 0.05; one-way ANOVA followed by Tukey test). Each column represents the mean, and vertical bars indicate SEM (*n* = 3 samples).

To assess the effects of treatment with DADS and GYY4137 in the central and peripheral antioxidant system, the protein levels of HO-1, NQO1, SOD-1, and GSTM1 in the MS and DRG were assessed. In the MS, sciatic nerve injury decreased the protein levels of SOD-1 (*p* < 0.0104; one-way ANOVA vs. sham-operated animals treated with SS) (Figure 5D), and both treatments, DADS and GYY4137, reversed its down-regulation. Moreover, both H_2_S donors increased the HO-1 (*p* < 0.0068; one-way ANOVA vs. sham-operated and nerve-injured animals administered with SS) (Figure 5A) and GSTM1 levels (*p* < 0.0018; one-way ANOVA vs. sham-operated and nerve-injured mice administered with SS) (Figure 5E). No alterations in NQO1 levels were observed in any group (Figure 5B).

**Figure 6:**
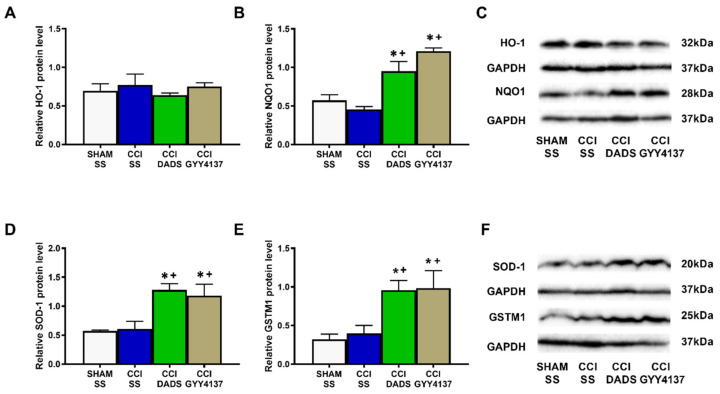
The influence of DADS and GYY4137 treatments on the HO-1, NQO1, SOD-1, and GSTM1 expression in the DRG of animals with neuropathic pain. Both treatments increased the expression of NQO1 (**B**), SOD-1 (**D**), and GSTM1 (**E**) in the DRG of mice with nerve injury-provoked neuropathic pain. No variations in HO-1 levels were identified (**A**). We used sham-operated (SHAM) mice treated with SS as a control group. All proteins are expressed in relation to GAPDH levels. Blots for HO-1 and NQO1 (**C**), as well as for SOD-1 and GSTM1 (**F**), are displayed. In all pictures, * symbolizes significant differences vs. SHAM-animals treated with SS and + vs. CCI-mice injected with SS (*p* < 0.05; one-way ANOVA followed by Tukey test). Each column represents the mean, and vertical bars indicate SEM (*n* = 3 samples).

In the DRG, whereas no changes in the expression of HO-1 were manifested (Figure 6A), both DADS and GYY4137 increased the expression of NQO1 (*p* < 0.0005; one-way ANOVA vs. sham-operated and nerve-injured animals injected with SS; Figure 6B), SOD-1 (*p* < 0.0092; one-way ANOVA vs. sham-operated and nerve-injured mice injected with SS; Figure 6D), and GSTM1 (*p* < 0.0179; one-way ANOVA vs. sham-operated and nerve-injured mice injected with SS; Figure 6E).

### 3.4. Effects of Treatment with DADS and GYY4137 on the Protein Levels of 4-HNE and BAX in the MS and DRG of Nerve-Injured Mice

**Figure 7:**
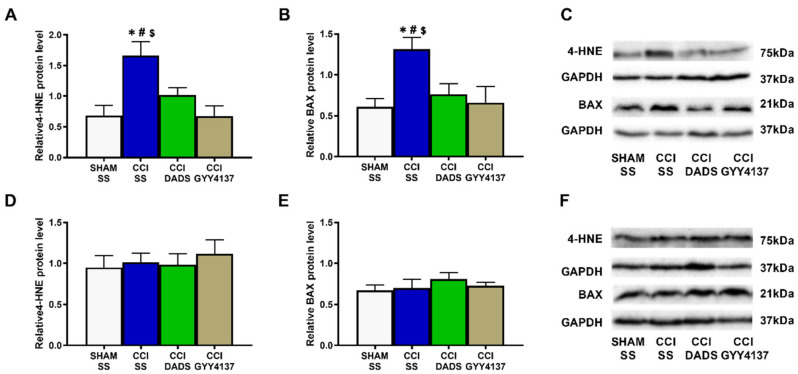
The influence of DADS and GYY4137 treatments on the expression of 4-HNE and BAX in the MS and DRG of mice with neuropathic pain. In the MS, both treatments regularized the overexpression of 4-HNE (**A**) and BAX (**B**). No changes in the expression of 4-HNE (**D**) or BAX (**E**) were detected in the DRG. Sham-operated (SHAM) mice treated with SS were used as control groups. 4-HNE and BAX are expressed relative to GAPDH levels. Blots for 4-HNE and BAX in the MS (**C**) and DRG (**F**) are presented. In all pictures, * symbolizes significant changes vs. SHAM-animals treated with SS; # vs. CCI-mice injected with DADS; and $ vs. CCI-mice injected with GYY4137 (*p* < 0.05; one-way ANOVA followed by Tukey test). Each column represents the mean, and vertical bars indicate SEM (*n* = 3 samples).

In the MS, nerve injury up-regulated the expression of 4-HNE (*p* < 0.0115; one-way ANOVA vs. sham-operated SS-injected animals; Figure 7A) and BAX (*p* < 0.020; one-way ANOVA vs. sham-operated SS-injected animals; Figure 7B), and both H_2_S donors stabilized them. No changes in the 4-HNE or BAX levels were noted in the DRG of nerve-injured animals injected with SS, DADS, or GYY4137 (Figure 7D,E).

### 3.5. Actions of DADS and GYY4137 Treatments on the Protein Levels of MOR and DOR in the MS and DRG of Nerve-Injured Animals

**Figure 8:**
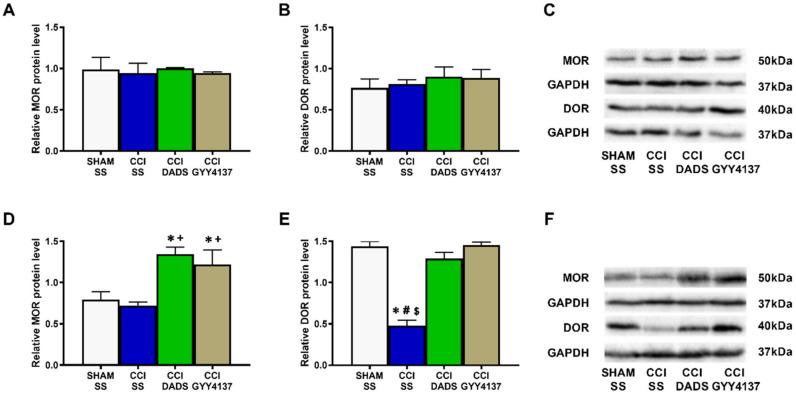
The influence of DADS and GYY4137 treatments on the expression of MOR and DOR in the MS and DRG of mice with neuropathic pain. Both treatments did not alter the protein levels of MOR (**A**) and DOR (**B**) in the MS, increased the expression of MOR (**D**), and avoided the down-regulation of DOR (**E**) in the DRG. Sham-operated (SHAM) mice treated with SS were used as control groups. MOR and DOR levels are expressed in relation to GAPDH. Blots for MOR and DOR in the MS (**C**) and DRG (**F**) are presented. In all pictures, * denotes significant changes vs. SHAM-animals injected with SS; + vs. CCI-mice injected with SS; # vs. CCI-mice injected with DADS; and $ vs. CCI-mice injected with GYY4137 (*p* < 0.05; one-way ANOVA followed by Tukey test). Each column represents the mean, and vertical bars indicate SEM (*n* = 3 samples).

The plausible mechanism implicated in the increased analgesic actions of morphine and UFP-512, induced by their co-treatment with H_2_S donors, was evaluated by studying the expression of MOR and DOR in the MS and DRG of sciatic nerve-injured mice treated with DADS or GYY4137. Our data showed that no alterations in the MOR (Figure 8A) or DOR (Figure 8B) levels were revealed in the MS of nerve-injured animals injected with SS, DADS, or GYY4137. In contrast, both H_2_S donors enhanced the protein levels of MOR (*p* < 0.0093; one-way ANOVA vs. sham-operated and nerve-injured animals injected with SS; Figure 8D), and normalized the decreased levels of DOR provoked by injury (*p* < 0.0001; one-way ANOVA vs. sham-operated animals injected with SS; Figure 8E) in the DRG of animals with neuropathic pain.

## 4. Discussions

In this study, we have shown that treatment with DADS or GYY4137 enhances the antinociceptive effects of MOR and DOR agonists, and increases their expression in the DRG of mice with neuropathic pain. Our data also revealed that the antiallodynic and antihyperalgesic effects of DADS and GYY4137 are reversed with specific MOR and DOR antagonists. Furthermore, both H_2_S donors modulate the oxidative and/or apoptotic reactions triggered by nerve injury in the MS and DRG of mice.

Chronic pain therapy is complex, basically due to the limited efficacy of conventional pharmacological treatments, especially with opioids [13], so increasing their efficacy is a potential strategy to improve the management of patients with neuropathic pain. Our study reveals that the combined administration of low doses of H_2_S donors with MOR or DOR agonists significantly improves the systemic and local antiallodynic and antihyperalgesic actions of morphine and UFP-512 in neuropathic pain. Our results further reveal that, while the co-treatment of DADS or GYY4137 with morphine or UFP-512 fully prevents CCI-induced mechanical and cold allodynia, these drug combinations do not completely reverse the thermal hyperalgesia provoked by nerve injury, thus revealing the greater effectiveness of these drug combinations in inhibiting allodynia than hyperalgesia caused by nerve-injury. Furthermore, because all tested combinations completely blocked mechanical and cold allodynia, and considering the few adverse effects induced by the local administration of opioids [14], the combination of local opioids with systemic H_2_S donors offers a safer and more effective strategy for the treatment of neuropathic pain. These data agree with the significant improvement of the local painkiller actions of DOR agonists in mice with inflammatory pain pre-treated with H_2_S donors [20]. Interestingly, other studies have demonstrated that the co-treatment of a carbon monoxide (CO)-slow-releasing compound, tricarbonyldichlororuthenium(II)dimer (CORM-2), or the HO-1-inducer, cobalt protoporphyrin IX (CoPP), increases the analgesic effects of systemically or locally administered morphine, but not those produced by DOR agonists during neuropathic pain [29]. These results underline the opposed actions induced by both gaseous neurotransmitters on the activation of DOR in the same pain model. Furthermore, our data indicate that both DADS and GYY4137 augment the peripheral expression of MOR and avoid the decreased levels of DOR observed in the DRG of nerve-injured mice, thus explaining the enhanced analgesic actions of morphine and UFP-512 in nerve-injured mice pre-treated with H_2_S donors. These results are consistent with the increased expression of DOR-induced H_2_S in the paws of mice with peripheral inflammation [20], and with the improved expression of MOR in the DRG of CCI-animals administered with CORM-2 or CoPP [29]. The lack of effects of CO/HO-1 activators in the expression of DOR in the DRG might explain the differing effects induced by H_2_S and CO on the painkilling actions of DOR agonists under neuropathic pain conditions. Our findings might be of great relevance in clinical practice, by allowing the use of opioids combined with H_2_S donors for the management of neuropathy. Moreover, and considering the lowered adverse effects of DOR agonists, its combination with DADS or GYY4137 should be considered as one interesting option to treat neuropathic pain.

Several studies have revealed that CO activates the synthesis of endogenous opioid peptides in inflammatory pain [35]. In this regard, our data demonstrate the reversion of the analgesic actions of both H_2_S donors with the systemic or local administration of MOR and DOR antagonists, naloxone and naltrindole. These results suggest the participation of the endogenous opioid system in the antiallodynic and antihyperalgesic effects induced by slow-releasing H_2_S donors during neuropathic pain. Our findings are consistent with the reversion of the antinociceptive actions of inhaled H_2_S with the administration of MOR antagonists in rats with diabetic neuropathy [36], and with the blockade of the analgesia induced by Na2S, another H_2_S releaser, with pre-treatment with selective antisense oligodeoxynucleotide probes against DOR and MOR [37]. This suggests that β-endorphins and enkephalins may be involved in the analgesic actions of H_2_S donors during nerve injury-induced neuropathic pain in mice. These findings indicate that the potentiation of the analgesic effects of morphine and UFP-512 produced by H_2_S might be exerted not only by enhancing the expression of MOR and DOR, but also by activating the endogenous opioid system in the CNS and PNS.

Oxidative stress participates in the progression of sensitization and cell apoptosis during neuropathic pain [38,39]. Indeed, elevated levels of 4-HNE, an endogenous α,β-unsaturated aldehyde generated during oxidative stress [40], were found in animals with carrageenan-induced inflammation [41] and with spinal cord injury-induced neuropathic pain [42]. Other studies have further revealed that the injection of 4-HNE incites mechanical allodynia [43], and that an accumulation of 4-HNE may disrupt many cell signaling pathways, including the regulation of apoptosis [40,44]. Moreover, increased levels of 4-HNE and/or BAX have been detected in the AMG and PAG of nerve-injured animals, and in the AMG of animals with osteoarthritis pain [23,24]. In accordance with these data, we observed high levels of 4-HNE and BAX in the MS of sciatic nerve-injured mice, which were normalized with DADS and GYY4137 treatments, therefore revealing the antioxidant and antiapoptotic effects of both H_2_S donors in the MS of mice with nerve injury-induced neuropathic pain. Both treatments further normalized the down-regulation of SOD-1 in the MS, and activated the expression of HO-1 and GSTM1 in this brain area, as well as the expression of NQO1, SOD-1, and GSTM1 in the DRG of nerve-injured mice. These results agree with the up-regulation of HO-1, NQO1, and GSTM1 provoked by GYY4137 in the AMG and PAG of nerve-injured animals [23], thus supporting the antioxidant properties of these H_2_S donors in the CNS and PNS of animals with neuropathic pain. Moreover, and considering the analgesic properties of several antioxidant compounds [45,46], we hypothesize that the antioxidant and antiapoptotic actions of DADS and GYY4137 may also contribute to potentiating the analgesic activity of MOR and DOR agonists during neuropathic pain.

Finally, it is well recognized that gut microbiota have a great impact in human health [47,48]. Recent studies have shown that neuropathic pain induced by nerve injury [49,50] or by long-term use of morphine [51] can lead to gut dysbiosis, by altering the composition of the microbiota [52], thereby impairing intestinal immune function, promoting neurodegenerative diseases such as Alzheimer and Parkinson [48,53,54], and aggravating the pain associated with neurodegenerative disease [55]. Other studies have also demonstrated that fecal microbiota transplantation and treatments with prebiotics and probiotics are potential therapeutic approaches for Alzheimer [56,57] and Parkinson [58,59], by modifying gut microbiota. Moreover, these treatments also relieve neuropathic pain by inhibiting inflammatory signals or modulating pro-inflammatory and anti-inflammatory T cells [60,61,62]. Furthermore, it is worth noting that the reconstitution of intestinal microbiota biofilm [63,64] is another important pathophysiological feature of H_2_S. Therefore, we postulate that the reversion of gut dysbiosis exerted by H_2_S might also contribute to their analgesic effects. Furthermore, the co-administration of low doses of H_2_S donors and opioids might also avoid the dysbiosis [51] and bacterial translocation [65,66] induced by high doses of opioids, thus maintaining the homeostasis of gut microbiota. Nevertheless, further studies are needed to demonstrate this hypothesis.

## 5. Conclusions

In summary, our results demonstrate an improvement in the analgesic properties of MOR and DOR agonists after their co-administration with slow-releasing H_2_S donors in animals with neuropathic pain, and suggest that these effects could be explained by the peripheral up-regulation of MOR and DOR, and the activation of the endogenous opioid system induced by DADS and GYY4137. In addition, H_2_S-induced antioxidant and antiapoptotic effects in the CNS and PNS could also contribute to potentiating the analgesic effects of opioids during neuropathic pain. This study proposes the co-treatment of H_2_S donors with MOR or DOR agonists as a potential therapy for neuropathic pain.

## Data Availability

Data is contained within the article or Supplementary Materials.

## Supplementary Materials

The following supporting information can be downloaded at: https://www.mdpi.com/article/10.3390/antiox11071321/s1, Figure S1: The inhibitory effects induced by the subplantar administration of UFP-512 on the mechanical allodynia, thermal hyperalgesia and cold allodynia induced by nerve-injury in mice.
