# Supplementary material for: Hydrogen Sulfide Increases the Analgesic Effects of µ- and δ-Opioid Receptors during Neuropathic Pain: Pathways Implicated
Source: Antioxidants (Basel). 2022 Jul 4;11(7):1321. doi: 10.3390/antiox11071321 (PMC9311550; doi:10.3390/antiox11071321)
Supplement: Supplementary file 1 [file antioxidants-11-01321-s001.zip › antioxidants-1767701-supplementary.pdf]

antioxidants

MDPI

We investigated the inhibition of the mechanical allodynia, thermal hyperalgesia and cold allodynia produced by the acute administration of different doses of UFP-512 (6, 12.5, 25, 50, and 100 µg/30 µl) at 30 days after surgery (n = 6 animals per dose). Mice were tested 1 h after UFP-512 injection, in accordance with our previous study [20].

The subplantar administration of UFP-512 inhibited the mechanical allodynia (Figure S1A), thermal hyperalgesia (Figure S1B) and cold allodynia (Figure S1C) in a dose-dependent manner and exerted the maximal effect at a dose of 100 µg. Moreover, the administration of UFP-512 at doses of 12.5, 25, 50, and 100 µg produced significantly greater inhibition of the mechanical allodynia, thermal hyperalgesia, and cold allodynia than those produced by saline ($p < 0.001$; one-way ANOVA). Additionally, high doses of UFP-512 (25, 50 and 100 µg) produced greater antiallodynic and antihyperalgesic effects than low doses (6 µg). Regarding the mechanical allodynia, UFP-512 at doses of 25, 50, and 100 µg produced greater inhibitory effects than dose produced by 12.5 and/or 25 µg ($p < 0.001$; one-way ANOVA). The inhibition of thermal hyperalgesia induced by UFP at 100 µg was significantly greater than those produced by 12.5, and 25 µg ($p < 0.001$; one-way ANOVA). Finally, the inhibition of cold allodynia induced by 100 µg of UFP-512 was significantly higher than those produced by 12.5, 25 and 50 µg of this drug.

The subplantar administration of UFP-512 did not have any effect on mechanical allodynia, thermal hyperalgesia, or cold allodynia in sham-operated mice (data not shown).

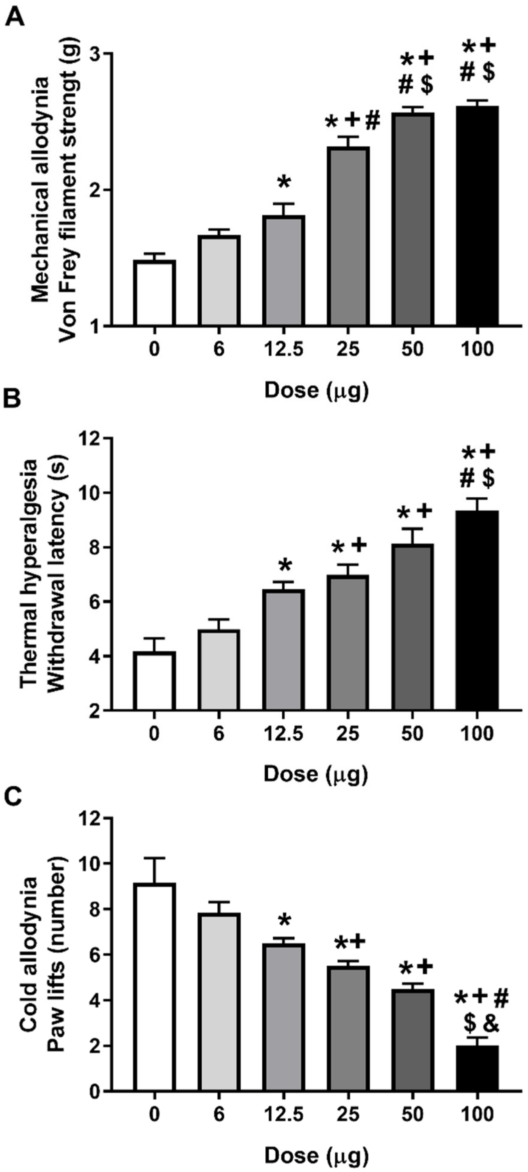

Figure S1. The inhibitory effects induced by the subplantar administration of UFP-512 on the mechanical allodynia, thermal hyperalgesia and cold allodynia induced by nerve-injury in mice. Effects of different doses of UFP-512 on the mechanical allodynia (A), thermal hyperalgesia (B), and cold allodynia (C) induced by sciatic nerve injury in the ipsilateral paw of mice. For each test, * represents significant differences vs. sciatic nerve-injured mice treated with saline (0 µg), + vs. sciatic nerve-injured mice treated with 6 µg of UFP-512; # vs. sciatic nerve-injured mice treated with 12.5 µg of UFP-512; $ vs. sciatic nerve-injured mice treated with 25 µg of UFP-512 and & vs. sciatic nerve-injured mice treated with 50 µg of UFP-512 ($p < 0.05$; one-way ANOVA followed by Tukey test). Data are expressed as mean values ± SEM; n = 6 animals per dose.
